# N-Doped Carbon NanoWalls for Power Sources

**DOI:** 10.1038/s41598-019-43001-3

**Published:** 2019-04-30

**Authors:** Stanislav A. Evlashin, Yurii M. Maksimov, Pavel V. Dyakonov, Andrey A. Pilevsky, Konstantin I. Maslakov, Yuri A. Mankelevich, Ekaterina N. Voronina, Sergei V. Vavilov, Alexander A. Pavlov, Elena V. Zenova, Iskander S. Akhatov, Nikolay V. Suetin

**Affiliations:** 1 Center for Design Manufacturing & Materials, Skolkovo Institute of Science and Technology, 3 Ulitsa Nobelya, Moscow, 121205 Russia; 2 Department of Chemistry, Lomonosov Moscow State University, 1-3 Leninskiye Gory, Moscow, 119991 Russia; 3 Skobeltsyn Institute of Nuclear Physics, Lomonosov Moscow State University, 1(2) Leninskiye Gory, Moscow, 119991 Russia; 4 Faculty of Physics, Lomonosov Moscow State University, 1-2 Leninskie Gory, Moscow, 119991 Russia; 5 Center for Energy Science and Technology, Skolkovo Institute of Science and Technology, 3 Ulitsa Nobelya, Moscow, 121205 Russia; 6 Moscow Institute of Physics and Technology, 9 Institutsky pereulok, Dolgoprudny, 141701 Russia; 7 Institute of microelectronics and nanotechnology, Russian Academy of Science, 32 A Leninsky Prospekt, Moscow, 119991 Russia

**Keywords:** Supercapacitors, Fuel cells

## Abstract

Cycling stability and specific capacitance are the most critical features of energy sources. Nitrogen incorporation in crystalline carbon lattice allows to increase the capacitance without increasing the mass of electrodes. Despite the fact that many studies demonstrate the increase in the capacitance of energy sources after nitrogen incorporation, the mechanism capacitance increase is still unclear. Herein, we demonstrate the simple approach of plasma treatment of carbon structures, which leads to incorporation of 3 at.% nitrogen into Carbon NanoWalls. These structures have huge specific surface area and can be used for supercapacitor fabrication. After plasma treatment, the specific capacitance of Carbon NanoWalls increased and reached 600 F g^−1^. Moreover, we made a novel DFT simulation which explains the mechanism of nitrogen incorporation into the carbon lattice. This work paves the way to develop flexible thin film supercapacitors based on carbon nanowalls.

## Introduction

Carbon NanoWalls (CNWs) were discovered several decades ago and now they show a certain potential for fabrication of various-purpose devices. Despite the fact that many properties of these materials are already well-known, new areas of application keep emerging^1,2^. CNWs are characterized by high specific surface area and can be used in fabrication of supercapacitors, hydrophobic surface coatings, field emitters and other products. Earlier, our team published a paper on electrochemical activation of CNWs synthesized by Direct Current Plasma Enhanced Chemical Vapor Deposition (DC PECVD)^3^. The electrochemical activation led to an increase in electrode specific capacitance from 105 F g^−1^ to 120 F g^−1^ ^4^. The composite electrode based on CNW@Si@CNW or CNW@Ge@CNW shows good stability and can be used to produce thin-film Li-ion batteries^5,6^, fabricate fuel cells^7^ or serve as a template for other catalytic materials^8^.

The most promising field of application for this material is electrodes for thin-film supercapacitors, which have become more popular recently due to ubiquitous adoption of wearable electronics and portable devices^9,10^. Crucial properties of CNWs are their high specific surface area, high electrical conductivity along graphite layers, and a total thickness of less than 5 µm. CNWs can be synthesized on the surface of different metals, semiconductors, carbon materials such as carbon paper and carbon fiber, thus allowing to fabricate flexible energy sources^11^. However, despite the fact that specific surface area of CNW amounts up to 1000 m^2^ g^−1^ ^5^, its specific capacitance reaches values of ~120 F g^−1^ ^4^. In order to increase specific capacitance of carbon materials, various metals oxide decoration methods are applied and, as a result, specific capacitance values of the structures are electrochemically enhanced by several times^12,13^. The decoration process requires additional technological procedures during or after the synthesis process. Recently, various nitrogen incorporation processes during the synthesis of graphene were demonstrated to have a positive effect on its specific capacitance due to an appearance of additional redox reactions^14^. One of these post-modification techniques, plasma treatment, was demonstrated to be able to produce a modified graphene with a specific capacitance of 855 F g^−1^ ^15,16^. Due to the fact that carbon materials possess chemically and structurally tunable properties, various methods of modification during the material synthesis stage have been proposed. These techniques include chemical vapor deposition and chemical tuning^17,18^.

In this paper, we investigate the electrochemical performance of CNWs after plasma modification. We report an increase in specific capacitance by a factor of 4.5 after the plasma-enhanced DC glow-discharge modification. Since CNW morphology is easy to modify with extensive plasma-enhanced post treatment, the reported technique can be used to produce energy storage devices.

## Experimental

### Materials synthesis

Rectangular glassy carbon electrodes (*A* = 0.5 cm^2^) were used as substrates for carbon nanowalls growth. The synthesis was carried out using DC PECVD in CH_4_ and H_2_ atmosphere^2,8^. This method resulted in thin CNW films on glassy carbon. We estimated the areal loading of CNW on glassy carbon substrates by growing films on large substrates (*A* = 4 cm^2^) and weighing. Thickness of the film was estimated from SEM views to be 2 μm. CNWs modification was performed in DC glow discharge with cylindrical cathode. Discharge voltage and current were 2 kV and 80 mA, respectively. Nitrogen pressure during the CNW treatment was 0.02 Torr. Modifications took place during first 2 hours. After 2 hours, we noticed no further changes in Raman spectra.

### Material characterization

Electrochemical studies were performed in 1 M H_2_SO_4_ solution on Advanced Electrochemical System Parstat 2273. Glassy carbon electrodes with CNW films were used as working electrodes in an asymmetric scheme with a large-area Au counter electrode and a standard hydrogen reference electrode. Structural analysis was performed with a use of Scanning Electron Microscope (SEM) Carl Zeiss Supra 40, Thermo Scientific DXR Raman Microscope spectrometer. X-ray photoelectron spectra (XPS) were recorded on an Axis Ultra DLD spectrometer (Kratos) using monochromatic Al Kα radiation.

### Computer modeling

DFT simulations were carried out with VASP (Vienna Ab initio Simulation Package) code^19^ and ORCA software^20,21^. GGA approximation with PBE exchange-correlation functional was applied for a larger model of graphene flake (72 and 120 atoms) to simulate potential chemical reactions, induced by N and O atoms, and hybrid B3LYP functional was used for a smaller model (~30 atoms) to estimate XPS shift for N-containing functional groups.

## Results

**Figure 1:**
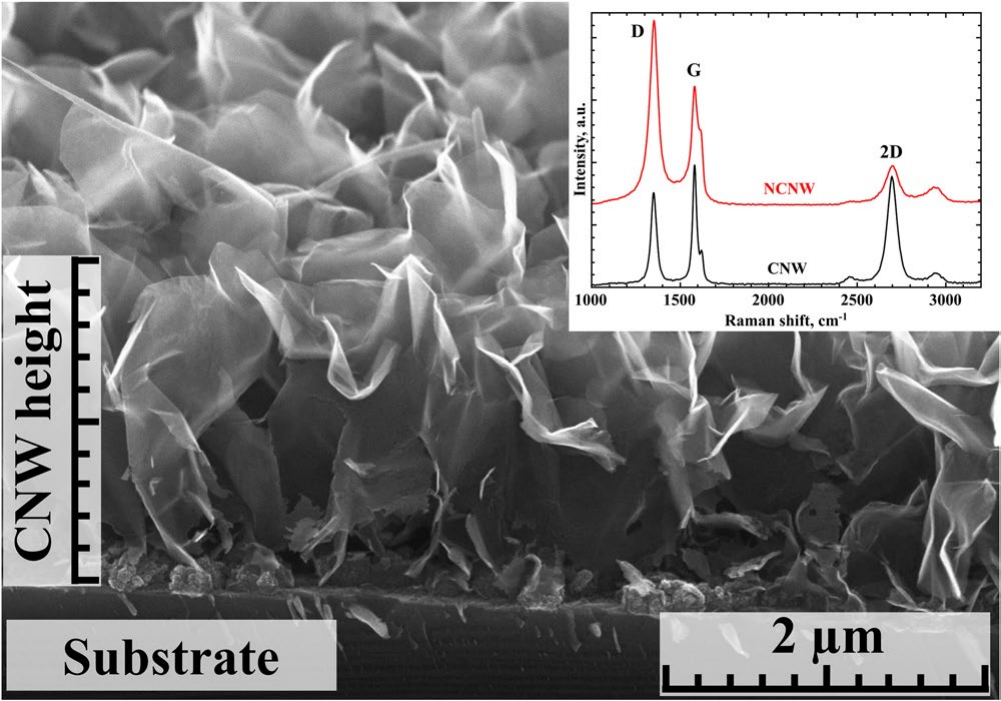
SEM image of CNW film. Top right inset shows Raman spectra of raw CNWs and CNWs after plasma modification in DC glow-discharge. D, G and 2D bands are indicated.

A SEM image of CNWs is presented in Fig. 1.

For the raw materials, the I(D)/I(G) ratio is 0.78, while the I(2D)/I(G) is 0.90. After 2 hours of plasma treatment, these values changed to 1.32 and 0.60, respectively (Fig. 1 inset). The shift of the ratios implies that the CNW structure has changed.

**Figure 2:**
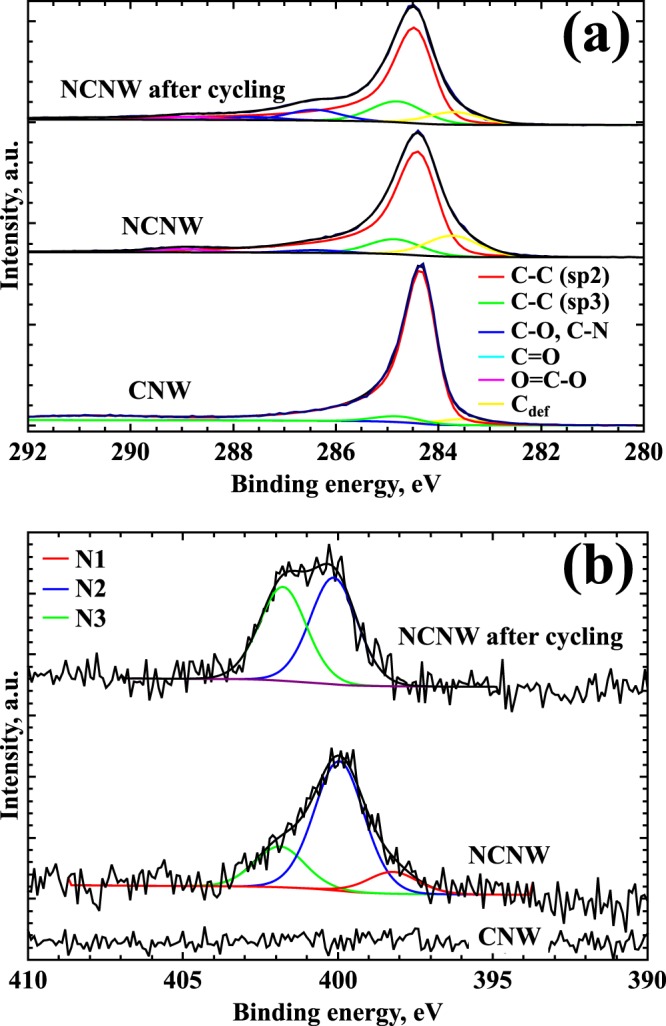
High resolution C1s (**a**) and N1s (**b**) XPS spectra of CNWs, NCNWs and NCNWs after cycling voltammetry.

To reveal the chemical state of carbon and nitrogen atoms, as-deposited CNWs, CNWs treated by the N_2_ plasma (further referred as NCNWs), and NCNWs after electrochemical cycling were analyzed by XPS (Fig. 2). XPS was preferred to CHNS in order to gain insight into atomic bonds and not only atomic content, and the penetration depth of XPS was sufficient for 2 μm thin films. The C1s XPS spectrum of CNWs contains the main contribution from sp^2^ carbon (284.5 eV) and a small peak at 284.9 eV that may be assigned to sp^3^ carbon. The oxygen content in this sample is ~2 at.%, while no nitrogen was detected by XPS. After plasma modification, the oxygen content sharply increases up to ~29.7 at.% and the nitrogen peak appears in the spectrum (Fig. 2b), corresponding to 2.4 at.% of nitrogen. This peak may be fitted by three components attributed to pyridinic nitrogen^22^ (N1, 0.3 at.%), pyrrolic or pyridonic nitrogen^16,22^ (N2, 2.4 at.%), and quaternary (graphitic) or oxidized pyridinic nitrogen species^22^ (N3, 0.5 at.%). In the C1s spectrum of this sample, the C_def_ peak at a binding energy lower than that of sp^2^ carbon is observed (Fig. 2a). This peak corresponds to nitrogen bonding to defect sites in sp^2^ carbon^22^. After 100 electrochemical cycles, the pyridinic (N1) peak disappears from the N1s spectrum while the content of N2 and N3 nitrogen species changes to 1.3 and 1.2 at.%, respectively. At the same time, the total nitrogen content only slightly changes after cycling (2.5 at.%). Cycling also increases the contribution of C−O bonds in the C1s spectrum at about 286.5 eV.

**Figure 3:**
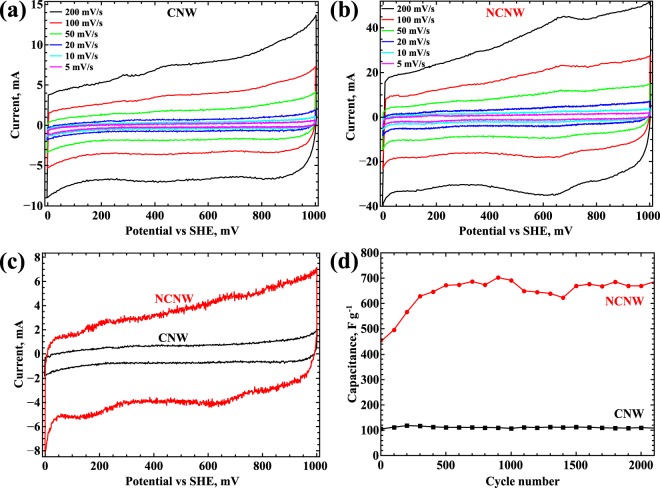
Cyclic voltammetry in 1 М H_2_SO_4_ electrolyte. (**a**) Electrochemical properties of CNWs before treatment, (**b**) NCNWs properties after plasma treatment, (**c**) and (**d**) cyclic voltammetry and cyclic stability of CNWs and NCNWs at scan rate of 20 mV s^−1^.

Electrochemical studies were performed in 1 M H_2_SO_4_ solution. Figure 3a shows cyclic voltammetry curves of CNWs for different scan rates. The same set of curves is presented for so-called NCNW samples. Specific capacitance was calculated as the area enclosed by the cyclic voltammetry curve, normalized by the potential range and the scan rate. A comparison of voltammetry curves is presented in Fig. 3c. Results show that glow-discharge plasma modification leads to an improvement in specific electrochemical capacitance from 104 F g^−1^ to 464 F g^−1^. These results are in a good agreement with work^4^. Thus, the plasma treatment process leads to an increase in specific capacitance of structures by a factor of 4.5. It can be assumed that such increase in capacitance correlates with oxygen content. However, according to another study, the increase of oxygen presence inside of the carbon structure doesn’t prominently affect the capacitance^4,23^. We attribute the increase in specific capacitance to nitrogen atom adsorption in bridge positions, as described below in the discussion section. N-doped CNWs demonstrate insignificant changes in specific capacitance with cycle number (Fig. 3d). We attribute this to slight changes in oxygen presence during cycling, despite a low potential range^23^.

## Discussion

CNW films placed on the anode of the dc discharge are subject to radical fluxes (N atoms), electrons and plasma radiation (no ion fluxes on the samples). Discharge current density at the cathode (j~0.4 mA cm^−2^) and measured cathode temperature T_c_ = 310 K hints at an abnormal regime of the discharge operation. Based on plasma-chemical and electron kinetics calculations of N_2_ plasma^24^ we estimate the electron and ion concentration *n*_e_ ≈ *n*_i_~10^8^ cm^−3^, reduced electric field E/N~55 T_d_ and respective electron temperature *T*_e_~1 eV in positive column between the cathode and sample surfaces. In these conditions, N atoms in ground N(^4^S) and lowest metastable N(^2^D) and N(^2^P) states are effectively produced in N_2_ dissociation by electron impacts. Cathode beam electrons also contribute to N_2_ dissociation. Monte Carlo calculations of the beam electrons energy dissipation show that ~1/3 of the gained energy goes into N_2_ dissociation and the remaining part is mostly due to N_2_ ionization^3^.

Negative oxygen ions (e.g. O^−^ as a main ion in our conditions) and other N_x_O_y_ species are also produced in air dc discharge plasma and, in lesser degree, in nitrogen plasma (due to air admixture). Negative ions in anode sheath could gain the hyperthermal energies (of about and lower than typical anode sheath potentials ~15–20 eV) and induce only slow erosion of the CNW surface (removal or substitution of surface C atoms). The created C-vacancies and surface could be occupied by impinging N and O atoms to produce various N and O containing surface sites observed in the XPS spectra, e.g. pyridinic N (N bonded to two carbon atoms), graphitic N (N bonded to three carbon atoms, also called substituted N or quaternary N) where surface C atoms are substituted by N atoms, pyrrole N and nitrogen oxide sites^16^. Pyridinic N sites and some O sites could be responsible for alteration of the CNW specific capacitance and catalytic activity^15,16^. In recent study^16^, it was revealed that pyridinic N in nitrogen-doped graphitic carbons creates the active sites for the oxygen reduction reaction (ORR). Carbon atoms next to pyridinic N are suggested to be the active sites at which O_2_ molecules are adsorbed as the initial step of the ORR^16^. We cannot exclude that the additional surface oxidization could be related with these active sites. It should be also noted that these stable N and O contained sites could be also removed by energetic O^−^ ions.

In our N_2_ (with air admixture) and air plasmas, O^−^ ions are mainly produced in dissociative attachment reaction

1 $${{\rm{O}}}_{2}+{\rm{e}}\to {{\rm{O}}}^{-}+{\rm{O}}$$

Here O_2_ molecules in ground (O_2_(X^3^Σ_g_^−^)) and lowest singlet (O_2_(a^1^Δ)) states will contribute in O^−^ production. O^−^ ions are destructed at the anode and associative attachment reactions

2 $${{\rm{O}}}^{-}+{\rm{O}}\to {{\rm{O}}}_{2}+{\rm{e}}$$

3 $${{\rm{O}}}^{-}+{{\rm{O}}}_{2}({{\rm{a}}}^{1}{\rm{\Delta }})\to {{\rm{O}}}_{3}+{\rm{e}}$$

4 $${{\rm{O}}}^{-}+{\rm{N}}\to {\rm{NO}}+{\rm{e}}$$

5 $${{\rm{O}}}^{-}+{\rm{NO}}\to {{\rm{NO}}}_{2}+{\rm{e}}$$

The concentrations of O^−^ ions in positive column and anode layer will be determined by a balance of all these processes.

**Figure 4:**
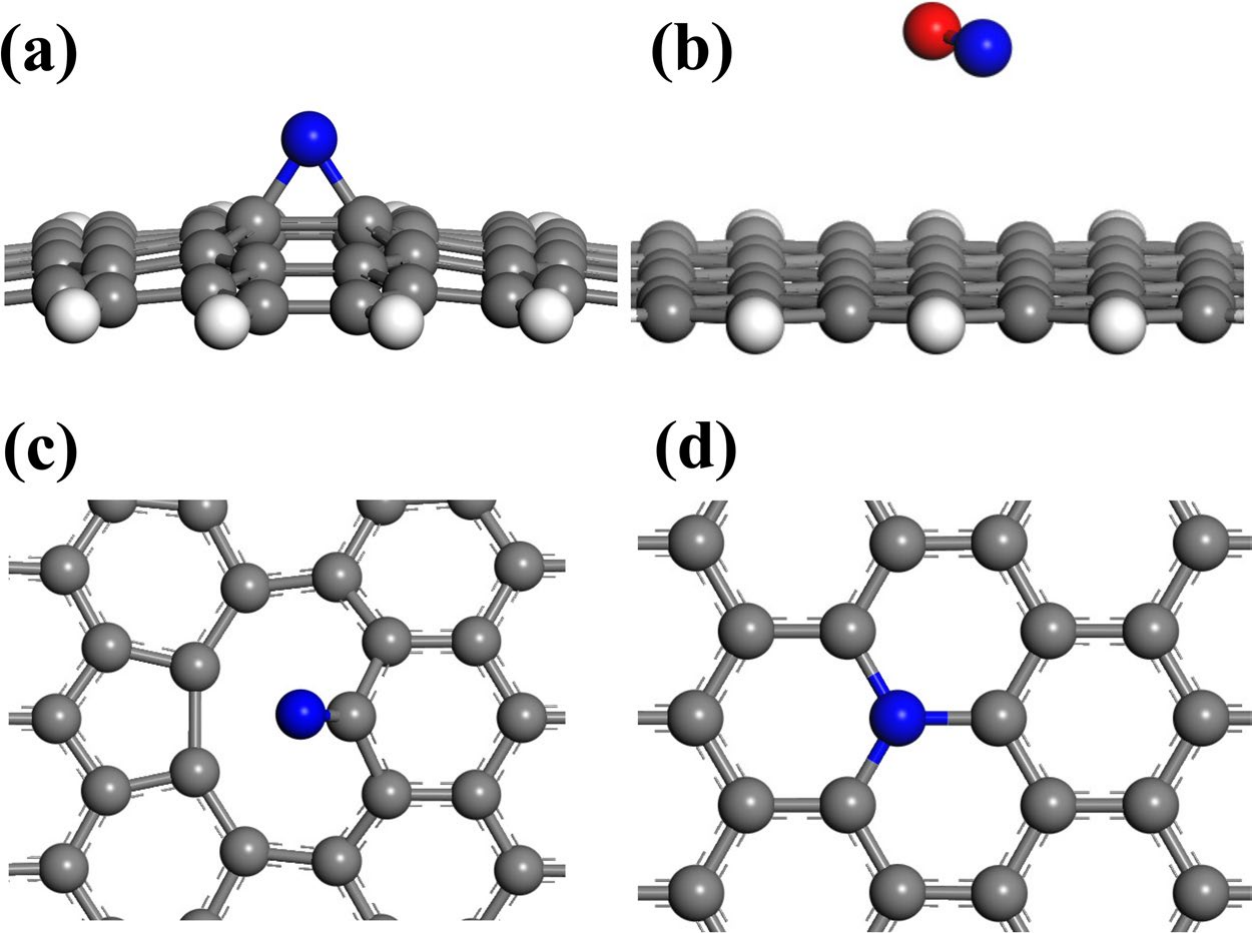
(**a**) N atom adsorbed in bridge position in the center of graphene flake; (**b**) the formation of a volatile NO species due to the O atom interaction with the bridge N site; (**c**) an N atom embedded into the carbon network; (**d**) an N atom adsorbed at the C-vacancy. C, H, N and O atoms are shown as grey, white, blue and red circles, respectively.

Possible reactions of N and O atoms on the created N and O sites of CNW surface were studied by density functional theory (DFT) method (the details of similar DFT modeling of N atoms interactions with low-k SiOCH films are described^25^). N(^4^S) atoms do not induce chemical reactions on regular graphene sheets, DFT calculations show only physical adsorption of an N(^4^S) atom above the graphene plane without chemical bonding with C atoms. In contrast to (N^4^S), metastable (N(^2^D) and N(^2^P)) atoms produce C-N-C bridge groups (Fig. 4a). However, these bridge N-states can be easily removed by oxygen or nitrogen atoms/ions (Fig. 4b):

6 $${{\rm{N}}}_{{\rm{bridge}}}+{{\rm{O}}}_{{\rm{gas}}}\to {{\rm{NO}}}_{{\rm{gas}}}$$

7 $${{\rm{N}}}_{{\rm{bridge}}}+{{\rm{N}}}_{{\rm{gas}}}\to {{\rm{N}}}_{2{\rm{gas}}}$$

The potential erosion of CNW surfaces by energetic O^−^ ions facilitates the insertion of N atoms into C-vacancies leading to the formation of pyridinic (N1), pyrrolic (N2) and graphitic (N3) states. These states are tightly bound with neighboring carbon atoms and are very stable as compared with bridge C–N–C states. Our static DFT calculations revealed that only excited N atoms are able to embed into a C-vacancy substituting a missing C atom and restoring the hexagonal network (Fig. 4c). The similar mechanism can be responsible for the formation of pyridinic and pyrrol sites. A ground-state N(^4^S) atom can be adsorbed on the C atom with a dangling bond (Fig. 4d). The energy of this state is 7.2 eV higher, and a substantial reconstruction of the graphene flake is observed and indicated that the formation of such C–N bonding is not probable for a flat graphene surface. However, additional pathways for N(^4^S) incorporation should be studied with dynamic DFT calculations since we cannot exclude potential intersystem crossing and graphene surface reconstruction.

The obtained results can explain the observed difference in nitrogen and oxygen content in the experimental XPS spectra as it was stated above (after N_2_ plasma exposure 2.4 at.% vs. 29.7 at. % for nitrogen and oxygen, respectively). In literature, 10–35 wt % of nitrogen contents (and similar surface N/C ratios) in nitrogen-doped carbon materials are reported in dependence on the carbonization temperature (e.g. ~500–1000 C) and relative independence on “the carbonaceous precursor, preparation conditions, and carbon type (porous carbon or graphene)”^26^. In our experiments, a rather different process of carbon surface enrichment by N atoms results in much lower percentage of surface N/C in CNWs at near room temperatures. The DFT calculations show the following principal problems of nitrogen (and oxygen) incorporation into CNWs under fluxes of thermal atoms. Even the most probable radical-radical addition reactions are not always feasible for ground states atoms N(^4^S). Excited atoms (e.g. metastable N(^2^D)) of lower concentrations are required for such reactions. In addition, the created single bond sites C-N or N bridge sites over regular graphene surface are easily destroyed under further impacts of atoms N and O resulting in volatile products N_2_ and NO, respectively. The production of stable nitrogen sites (N atoms binding with two or three surface C atoms) requires special and rare surface configurations (sites) e.g. C vacancies, two adjacent C vacancies at the CNW edges. The limited number of these appropriate sites (presented in as-deposited CNW films or created by hyperthermal O^−^ ions and O atoms) implies low percentage of stably incorporated nitrogen.

Diverse reactions of O atoms with various surface sites (including nitrogen sites) will be reported in a separate paper. In general, O atoms are more reactive with CNW surface than N atoms, and O atoms/ions are able to form more stable ether-like C–O–C bridge groups and initiate chemical reactions leading to the formation of defects (vacances) and volatile CO and CO_2_ molecules^27,28^. However, the observed high uptakes of oxygen (much higher than the fractions of incorporated nitrogen) are mainly related to *ex-situ* oxygen adsorption in ambient air. 7.4% ± 0.3% fractions of surface oxygen after N-implantation of HOPG at ion’s dosages of 10^15^–10^17^ ions/cm^2^ ^29^. The authors^29^ have explained this effect by “reactive surface defects (i.e., dangling bonds, step edges, radicals, etc.) created by the implantation process are immediately passivated and/or oxidized by oxygen species upon exposure to atmosphere”. In our DFT calculations, we have also observed the dissociation of O_2_ molecules on C-vacancies. Moreover, atmospheric molecular oxygen can dissociate at C-vacancies^30^, forming strong C–O and C=O bonds and thus increasing the oxygen content.

DFT calculations were also carried out to explain the obtained XPS spectra. We have calculated the thresholds of X-ray absorption spectra with B3LYP exchange-correlation functional; these thresholds are closely related to the shift in the XPS spectrum due to chemical bonding^31^. The difference between calculated shifts for pyridinic (N1) and graphitic (N3) groups is ~3.2 eV which coincides quite well with the experimental data (401.8–398.2 = 3.6 eV, see Fig. 2). When H atom interacts with pyridinic N site, an N–H bond forms, and the N atom becomes bound with three atoms. The calculated shift for such a configuration is ~2.6 eV higher than for the initial pyridinic N site indicating that H adsorption on this site leads to its conversion from pyridinic-N to graphitic-N or to pyrrolic-N in the XPS spectra. Such an effect was observed in^32^, when the exposure to hydrogen caused the disappearance of N1 peak and the enhancement of graphitic N3 peak. The similar shift could be induced by OH radical adsorption near a pyridinic site and its transformation into a pyridonic site^16^.

## Conclusion

In this study, we demonstrate a simple and efficient approach for surface modification of carbon materials. In particular, we used graphene derivative called Carbon NanoWalls, which are known for high specific surface area and can be used as active materials to produce electrochemical energy sources. As-deposited CNWs posses no nitrogen content. After CNWs treatment by N_2_ DC discharge plasma (CNWs were placed on anode and exposed to N atoms, electrons and negative ions), three forms of the incorporated nitrogen are detected by XPS: pyridinic, pyrrolic and graphitic sites. After electrochemical study of cyclic voltammetry in 1 M H_2_SO_4_ solution, we have detected the disappearance of pyridinic sites, minor changes in pyrrolic sites fraction and doubling of graphitic sites. DFT calculations were used to study possible production mechanisms for these nitrogen sites. Our research demonstrates the incorporation of nitrogen up to ~3 at.%, which leads to an increase in electrochemical specific capacitance by a factor of 4.5–6. The specific capacitance of Carbon NanoWalls at a scan rate of 20 mV s^−1^ reached ~600 F g^−1^, while that of non-modified structures was only 105 F g^−1^. We assume that the proposed technique can be used for effective post-treatment surface modification of carbonaceous materials.
